# The roles and applications of neural stem cells in spinal cord injury repair

**DOI:** 10.3389/fbioe.2022.966866

**Published:** 2022-08-29

**Authors:** Wen Guo, Xindan Zhang, Jiliang Zhai, Jiajia Xue

**Affiliations:** ^1^ Department of Orthopaedic Surgery, Peking Union Medical College Hospital, Chinese Academy of Medical Science and Peking Union Medical College, Beijing, China; ^2^ Beijing Laboratory of Biomedical Materials, Beijing University of Chemical Technology, Beijing, China; ^3^ Department of Orthopaedic Surgery, Peking Union Medical College Hospital, Beijing, China

**Keywords:** neural stem cell, spinal cord injury, repair, review, application

## Abstract

Spinal cord injury (SCI), which has no current cure, places a severe burden on patients. Stem cell-based therapies are considered promising in attempts to repair injured spinal cords; such options include neural stem cells (NSCs). NSCs are multipotent stem cells that differentiate into neuronal and neuroglial lineages. This feature makes NSCs suitable candidates for regenerating injured spinal cords. Many studies have revealed the therapeutic potential of NSCs. In this review, we discuss from an integrated view how NSCs can help SCI repair. We will discuss the sources and therapeutic potential of NSCs, as well as representative pre-clinical studies and clinical trials of NSC-based therapies for SCI repair.

## 1 Introduction

Spinal cord injury (SCI) is a devastating condition that disables patients and constitutes a considerable proportion of the global health burden. According to a globally-collaborated study, in 2016 there were 0.93 million new cases and 27.04 million prevalent cases of SCI worldwide (Collaborators, 2019). In China, unfortunately, the epidemiological data are insufficient and dispersed regionally (Yuan et al., 2018). The incidence of SCI in different regions of China ranges from 23.7 million to 60 per million (the latter in Beijing) (Reinhardt et al., 2017;Wei, 2007). Patients of SCI suffer mentally, physically, and economically. In Canada, the economic cost of a patient of traumatic SCI is $1.5 million for incomplete paraplegia and $3.0 million for complete tetraplegia (Krueger et al., 2013).

Managing SCI is difficult, due mainly to its complex pathophysiology and destructive outcomes. The pathophysiology of SCI can be divided into two phases (Rowland et al., 2008). The primary phase refers to the initial physical trauma to the spinal cord, directly disrupting blood spinal cord barriers. This causes hemorrhage, edema, and damage to local structures such as neurons, oligodendrocytes, blood vessels, and cell membranes (Ahuja et al., 2017; Tran et al., 2018). The subsequent secondary phase, induced by inflammation, refers to delayed tissue destruction involving vascular dysfunction, ischemia, edema, electrolyte shifts, excitotoxicity, inflammation, free radical injury, and apoptosis (Huang L. et al., 2021). While resident neural stem cells (NSCs) may play a key role in the regenerative process in SCI, endogenous repair in the injured spinal cord remains limited and poorly understood (Hachem et al., 2020). As a result, the normal functioning of the spinal cord is lost and is hard to restore.

The current management of SCI mainly focuses on alleviating secondary injuries and restoring normal neural functioning in both acute and subacute phases (Huang et al., 2020). The principal treatments include pharmacological neuroprotection, early surgical depression and stabilization, and arterial pressure augmentation (Karsy and Hawryluk, 2019). Other options such as cell transplantation therapies (with or without scaffolds) are being actively explored and have not yet been applied in clinical practice (Venkatesh et al., 2019). Currently, the effective clinical management of SCI remains limited (Donovan and Kirshblum, 2018) and patients continue to suffer physiologically, mentally, and financially from it.

Researchers are thus still struggling to find effective therapies for SCI repair. Stem cell-based transplantation therapies are considered promising, major options of which include mesenchymal stem cells (MSCs), NSCs, embryonic stem cells (ESCs), and induced-pluripotent stem cells (iPSCs) (Gong et al., 2020). Compared with other sources of stem cells, the unique feature of NSCs is the restricted differentiation of fates into neural lineages (Andreotti et al., 2019). This endows NSCs with a promising potential for a wide range of neurological diseases, including neurodevelopmental and neurodegenerative diseases, as well as SCI (Grochowski et al., 2018; De Gioia et al., 2020). Currently, NSCs are less popular than MSCs, mainly due to the availability of stem cells (Shao et al., 2019). However, the availability of NSCs has improved with the exploration of alternative sources of NSCs. In addition, NSCs are multipotent cells that give rise to neuronal and neuroglial lineages, making them suitable candidates for SCI repair (Huang L. et al., 2021). Although therapeutic outcomes vary with many factors, evidence has supported the positive efficacy of NSCs for SCI repair (Yousefifard et al., 2016). Therefore, we believe that NSCs possess considerable potential for SCI repair. There are many studies that focus on various aspects of NSCs, including the methodology to generate NSCs, possible repair mechanisms of NSCs, preclinical studies, and clinical trials to test validate therapies using NSCs (Stenudd et al., 2015; Yousefifard et al., 2016; Zhu et al., 2018; de Freria et al., 2021). Nonetheless, an integrated review is needed for a broader discussion of how NSCs may help with SCI repair.

We will first illustrate both the endogenous and exogenous sources of NSCs. We will then explain the therapeutic potential of NSCs and the mechanism for promoting SCI repair. Next, major preclinical strategies and clinical applications which have used the potential of NSCs in recent years will be summarized and discussed, with an emphasis on strategy design and outcomes. Finally, we will suggest major obstacles and future perspectives. We sincerely hope that this review may inspire future studies in this field.

## 2 Sources of NSCs

NSCs are multipotent stem cells that can self-renew and differentiate into neurons and neuroglial cells (Stenudd et al., 2015; Shoemaker and Kornblum, 2016; Andreotti et al., 2019). These features are the basic properties of NSCs no matter whether they are endogenous or exogenous. In this review, endogenous NSCs refer to those which reside in the injured spinal cord, considering that SCI is the main topic of this review. Exogenous NSCs, on the other hand, refer to NSCs which are prepared outside of the injured spinal cord.

### 2.1 Endogenous NSCs in the spinal cord

Endogenous NSCs in the spinal cord can be triggered by SCI (Grégoire et al., 2015). These consequently proliferate, migrate to the lesion site, and differentiate into a much larger number of astrocytes and a smaller number of oligodendrocytes, contributing to the glial scars. Endogenous NSCs also exert a neurotrophic effect and maintain tissue integrity during SCI (Stenudd et al., 2015).

Ependymal cells are the widest accepted endogenous NSCs in the spinal cord; their multipotency has been demonstrated *in vitro* in mice models*.* These cells are ciliated and line the central canal of the spinal cord. They are responsible for the propulsion of cerebrospinal fluid (CSF) and originate from radial glial cells via symmetrical division between Embryonic Day 13.5 (E13.5) and E15.5 (Ortiz-Álvarez et al., 2019). *In vitro* experiments have shown that ependymal cells could divide rigorously and manifest stem cell properties, giving rise to astrocytes, oligodendrocytes, and neurons (Barnabé-Heider et al., 2010). Unlike NSC niches in the brain, where sub-ependymal cells rather than ependymal cells more evidently manifest NSC properties (Chiasson et al., 1999), sub-ependymal cells in the spinal cord are rudimentary and lack the capability of cell proliferation (Hamilton et al., 2009). Although certain types of sub-ependymal cells in the spinal cord may manifest NSC properties (Sabourin et al., 2009; Foret et al., 2010), evidence is preliminary and not solid, especially in comparisson with ependymal cells. However, the close proximity of subependymal cells and ependymal cells may hinder the identification of specific cell types and create further debate. Nonetheless, due to the lack of evidence, ependymal rather than sub-ependymal cells are commonly considered NSCs in the spinal cord (Grégoire et al., 2015; Stenudd et al., 2015; Xue W. et al., 2021).

However, it is also debatable whether ependymal cells are endogenous NSCs in the spinal cord (Havelikova et al., 2022). This is largely because of the quiescent behavior of ependymal cells *in vivo*. In the intact spinal cord, ependymal cells are mainly non-proliferative and rarely differentiate into other cell types (Barnabé-Heider et al., 2010). Previous studies reported that ependymal cells can be activated after SCI in animal models (Moreno-Manzano et al., 2009; Lacroix et al., 2014; Ye et al., 2018). Cawsey et al. reported in 2015 that nestin-positive cells can be enriched after SCI in humans (Cawsey et al., 2015). Because nestin is massively expressed in NSCs, Cawsey et al. deduced that ependymal cells respond actively to SCI (Cawsey et al., 2015). However, the latest evidence in 2021 reported that nestin-positive cells enriched at SCI lesion sites are rarely ependymal cells in mice (Xue X. et al., 2021). Moreover, a study on 21 adults reported that the ependymal region in the spinal cord does not proliferate after SCI, further challenging the neurogenic capabilities of ependymal cells in the spinal cord (Paniagua-Torija et al., 2018).

**FIGURE 1:**
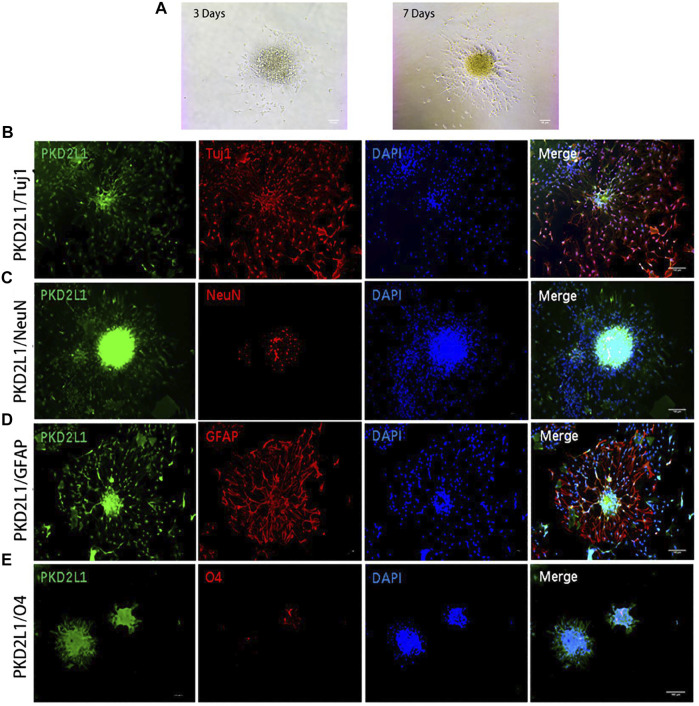
Neurospheres formed by CSF-cNs were tri-potent (Wang S. et al., 2021). **(A)** Differentiation of neurospheres after adherent culture on Days 3 and 7. **(B–E)** Immunofluorescence analysis showing that neurospheres differentiated into neurons that were positive for Tuj1 **(B)** and NeuN **(C)**, astrocytes that were positive for GFAP **(D)**, and oligodendrocytes that were positive for O4 **(E)**, respectively. Scale bar represents 50 μm in **(A)** and 100 μm in **(B–E).**

Confusion regarding the stem cell capabilities of ependymal cells persists. In 2018, a single-cell transcriptome research reported that ependymal cells in an intact spinal cord rarely manifest stem cell functionalities (Shah et al., 2018), creating further controversy. Therefore, researchers continue to search for endogenous stem cells in the spinal cord. CSF-contacting neurons (CSF-cNs) have recently been isolated, located near the central canal of the spinal cord. It is reported that PKD2L1^+^ CSF-cNs express NSC markers and manifest the differential capabilities of multiple neuronal and neuroglial cell lineages (Figure 1 (Wang S. et al., 2021)).

Although sources of endogenous NSCs in the spinal cord remain elusive, NSCs can still be extracted from the central canal region of the spinal cord. Refer to Galuta et al. (2020) for detailed protocols. Other well-documented cells in the spinal cord such as astrocytes, microglia, and oligodendrocytes fail to differentiate into other cell lineages (Buffo et al., 2008; Young et al., 2013) and thus fail to manifest the properties of NSCs.

### 2.2 Exogenous NSCs

Exogenous NSCs are harvested outside of injured spinal cords and are convenient for cell transplantation therapy. Like endogenous NSCs, these cells possess similar features: self-renewal, proliferation, and differentiation into multiple neuroectodermal lineages. *In vitro* identification of NSCs utilizes a neural colony-forming cell assay to assess their proliferative capabilities and immunocytochemical staining to assess differentiation capabilities into neuronal and neuroglial lineages. NSCs can be enriched by CD133+ markers with flow cytometry. *In vivo*, immunohistochemistry facilitates the identification of NSCs, markers of which include nestin (an intermediate filament protein expressed in the neuronal precursor cells of the subventricular zone), Sox2 (transcription factors essential for self-renewal), Ki67 (markers for active phases in the cell cycle), PCNA (proliferating cell nuclear antigen which participates in DNA replication and repair), MCM2 (minichromosome maintenance complex component 2, responsible for initiating and elongating DNA replication), and Musashi-1 (expressed in fetal and adult NSCs and which participates in self-renewal) (Siebzehnrubl et al., 2011).

The major sources of exogenous NSCs include direct extraction from CNS tissues or the induction of somatic cells via indirect or direct reprogramming. Exogeneous NSCs can be extracted directly from neurogenic niches in mammals—that is, the ependymal region in the spinal cord, the subgranular zone of the dentate gyrus, and the subventricular zone of lateral ventricles (Galvan and Jin, 2007; Lu, 2017). However, the accessibility of central nervous system (CNS) tissue restricts the application of such NSCs. Therefore, iPSC-derived NSCs and induced NSCs (iNSCs) by reprogramming somatic cells are increasingly preferable sources of exogenous NSCs, especially considering the convenience of production and the avoidance of ethical concerns (Hong et al., 2014; Grochowski et al., 2018).

Detailed protocols for generating human iPSC-derived NSCs are described by D’Aiuto et al. (2014). iPSC-derived NSCs are prepared by biasing differentiation fates of iPSCs with neural precursor selection mediums. After this step, characteristic neural rosettes and neurosphere-like structures can be formed and NSCs can thus be purified and selected. Current evidence has shown that human iPSC-derived NSCs possess neural stemness, having the capacity to differentiate into both neuronal and neuroglial lineages (López-Serrano et al., 2016; Lukovic et al., 2017). However, preliminary animal studies suggest that tumor formation is a serious problem caused by undifferentiated iPSCs (Ramotowski et al., 2019).

iNSCs are produced by direct reprogramming from somatic cells, bypassing the iPSC stage. Candidate somatic cells include astrocytes, fibroblast, and human umbilical cord blood cells (Corti et al., 2012; Hong et al., 2014; Kim et al., 2017; Zarei-Kheirabadi et al., 2019). Somatic reprogramming can be induced by specific transcription factors and/or pharmacological molecules (Erharter et al., 2019). Directly converted iNSCs can differentiate into both neuroglial and neuronal lineages, covering all cell types in the adult spinal cord and serving the role of NSCs (Hong et al., 2014). Compared with iPSC-derived NSCs, iNSCs have lower risk of tumorigenicity.

Exogenous NSCs can also be obtained from olfactory mucosa, the NSCs of which refer to basal cells, including horizontal and global basal cells (HBC and GBC) (Calvo-Ochoa et al., 2020). Horizontal basal cells are aligned as a monolayer above the basal lamina, expressing oligodendrocyte and astrocyte progenitor markers; they can differentiate into oligodendrocytes and Schwann cells *in vivo* (Ohnishi et al., 2013). Global basal cells, on the other hand, located superficially above the HBC layer, have been shown to remain quiescent in normal conditions and then activate upon epithelial injury to divide and differentiate to repair olfactory epithelium (Chen et al., 2014; Jang et al., 2014). NSCs from olfactory mucosa have been the subject of special attention because of their potential to avoid graft-versus-host disease when transplanted autologously. As for extraction, in 2020 Voronova et al. developed a protocol that can enrich NSCs from human olfactory mucosa, facilitating studies concerning transplantation therapies (Voronova A. D. et al., 2020). Preliminary results showed that transplanting NSCs from human olfactory mucosa into recipient rats with chronic SCI may improve the motor activity of hind limbs.

The enteric nervous system (ENS), which harbors enteric NSCs (ENSCs), can be considered another promising source of NSCs. ENSCs have the potential for autologous transplantation and can be harvested by routine gastrointestinal procedures such as endoscopy (Metzger et al., 2009a; Metzger et al., 2009b). Preliminary evidence from chicken models support the potential of ENSCs for SCI repair, since ENSCs can serve as NSCs and differentiate into both neural and neuroglial lineages (Jevans et al., 2018).

In summary, exogenous NSCs can be prepared using various methods. They were first extracted from embryonic/fetal CNS tissue, the protocols and therapeutic efficacy of which have been well-established (Nori et al., 2017). However, ethical concerns restrict the application of these types of NSCs. iPSC-derived NSCs and iNSCs are alternative sources of NSCs, allowing NSCs to be prepared from somatic cells. Convenience of production and the avoidance of ethical issues make these NSCs promising. Olfactory mucosa and ENS are novel sources for extracting NSCs; such technologies provide opportunities for autologous transplantation. However, considering the limited evidence, more studies are needed to not only optimize extraction protocols but also to validate therapeutic efficacy.

## 3 Therapeutic potential of NSCs in SCI

**FIGURE 2:**
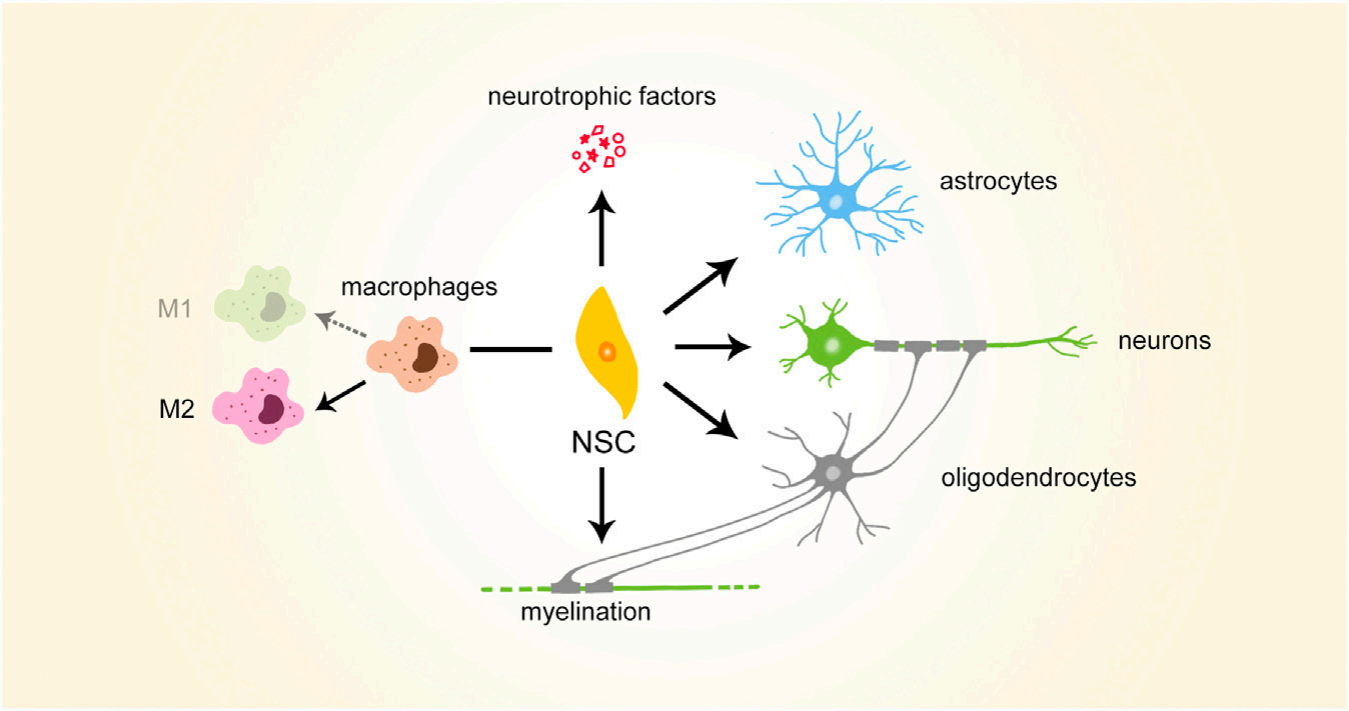
Brief illustration of therapeutic potential of NSCs. NSCs, neural stem cells

NSCs possess various therapeutic potentials which are beneficial to SCI repair (Figure 2). According to the process of SCI repair, NSCs can first modulate inflammatory and immune microenvironments, polarizing macrophages to anti-inflammatory M2 type. NSCs can then secrete growth factors to support motor and sensory axonal growth. In addition, they can differentiate into neuronal and neuroglial lineages, serving as cell sources for SCI repair. Biasing the differentiation fates of NSCs may help reduce glial scars formed in SCI repair. NSCs can also increase myelination during SCI repair, possibly via differentiation into oligodendrocytes.

### 3.1 Modulation of the inflammatory microenvironment

Mutual interactions exist between the inflammatory microenvironment of SCI and NSCs (Grégoire et al., 2015). Although many mysteries remain, the modulation of the inflammatory and immune microenvironment by NSCs may be a promising target for SCI repair. An *in vitro* co-culture study revealed that NSCs can induce macrophage polarization to M2 type, dependent on interleukin (IL)-4 (Ji et al., 2020). Compared with pro-inflammatory M1 macrophages, M2 macrophages are considered anti-inflammatory and reparative (Varga et al., 2016). However, during SCI, M2 macrophages deplete over time while M1 macrophages stay activated and elevated throughout the course, hindering natural repair after SCI (Gensel and Zhang, 2015). Therefore, macrophagic polarization mediated by NSCs serves as a potential therapeutic target for SCI repair.

### 3.2 Secretion of neurotrophic factors

NSCs can naturally secret neurotrophic factors involving glial cell line-derived neurotrophic factor (GDNF), nerve growth factor (NGF), vascular endothelial growth factor (VEGF), brain-derived neurotrophic factor (BDNF), and insulin-like growth factors (IGF)-1 (Lu et al., 2003; Oliveria et al., 2016). These factors support the axonal growth and vascular growth of the lesioned spinal cord, facilitating SCI repair. In addition, certain signals can induce NSCs to express some neurotrophic factors. Constitutive Raf-Erk activation induces NSCs to express soluble factors, including leukemia inhibitory factor (LIF), responsible for astrocyte differentiation (Rhee et al., 2016). Considering the beneficial effects of many factors, transfection technology can be used to modify NSCs to overexpress certain neurotrophic factors, such as neurotrophin 3 (NT-3), cells of which showed improved migration and preferred oligodendrocyte differentiation compared with controlled NSCs (Hwang et al., 2011).

### 3.3 Differentiation into neuronal and neuroglial lineages

NSCs can differentiate into multiple cell lineages, which is a promising therapeutic target; abundant studies have concentrated on creating advantaged microenvironments for NSC migration, proliferation, and/or differentiation. *In vivo*, endogenous NSCs predominantly differentiate into astrocytes, promoting glial scar formation and hindering neural regeneration (Götz et al., 2015). Hence, on the one hand, the blockade of the astrocyte differentiation may help with SCI repair. For example, C1q and C3a, produced by neutrophils, can induce migration of NSCs and their differentiation into astrocytes. Blockading these complement components reversed astrocyte differentiation and migration (Hooshmand et al., 2017). On the other hand, some mechanisms that can alter the differentiation fates of NSCs are also being explored and may be beneficial for SCI repair. For example, oligodendrocyte transcription factor 2 (OLIG2), the transcription factor of oligodendrocytes, is not naturally expressed in endogenous NSCs. However, if OLIG2 is engineered to be expressed in the ependymal cells in a mouse model, these cells will efficiently develop into oligodendrocytes (constituting about 30% of the ependymal progeny) and migrate into demyelinated sites (Llorens-Bobadilla et al., 2020).

Furthermore, the neuronal differentiation of NSCs is another alternative preferred differentiation fate, establishing a basis for reconstructing neural circuits in injured sites of the spinal cord. Complex signaling pathways and regulatory mechanisms are involved in NSC neuronal differentiation, such as the leukemia inhibitory factor (LIF) signaling pathway and the JNK/MAPK signaling pathway (Vieira et al., 2018). Related transcription factors, miRNAs, and ECM components can be targeted to induce the neuronal differentiation of NSCs, creating abundant action sites for SCI repair. More targets for neuronal differentiation are now emerging, broadening the possibilities for SCI repair strategies (Li et al., 2020a; Wu et al., 2020; Li T. et al., 2021).

### 3.4 Myelination promotion

NSCs can increase myelination in the injured spinal cord, possibly via migration into the cystic cavity and differentiation into oligodendrocytes (Sankavaram et al., 2019). Demyelination happens during SCI and impairs electronic signal conduction due to the loss of insulation. Research has shown that differentiated oligodendrocytes from NSCs will increase myelination locally and thus promote conduction, which favors SCI repair (Assinck et al., 2017). However, it remains debatable whether myelination from exogenous cells is a suitable strategy for SCI repair, given that precursor cells with oligodendrocyte fates did not always result in significant motor recovery after SCI (Plemel et al., 2011). Therefore, the exact benefits of myelination caused by transplantation of NSCs are still unclear.

## 4 Applications of NSC transplantation therapy to treat SCI

### 4.1 Progresses in preclinical tests

**FIGURE 3:**
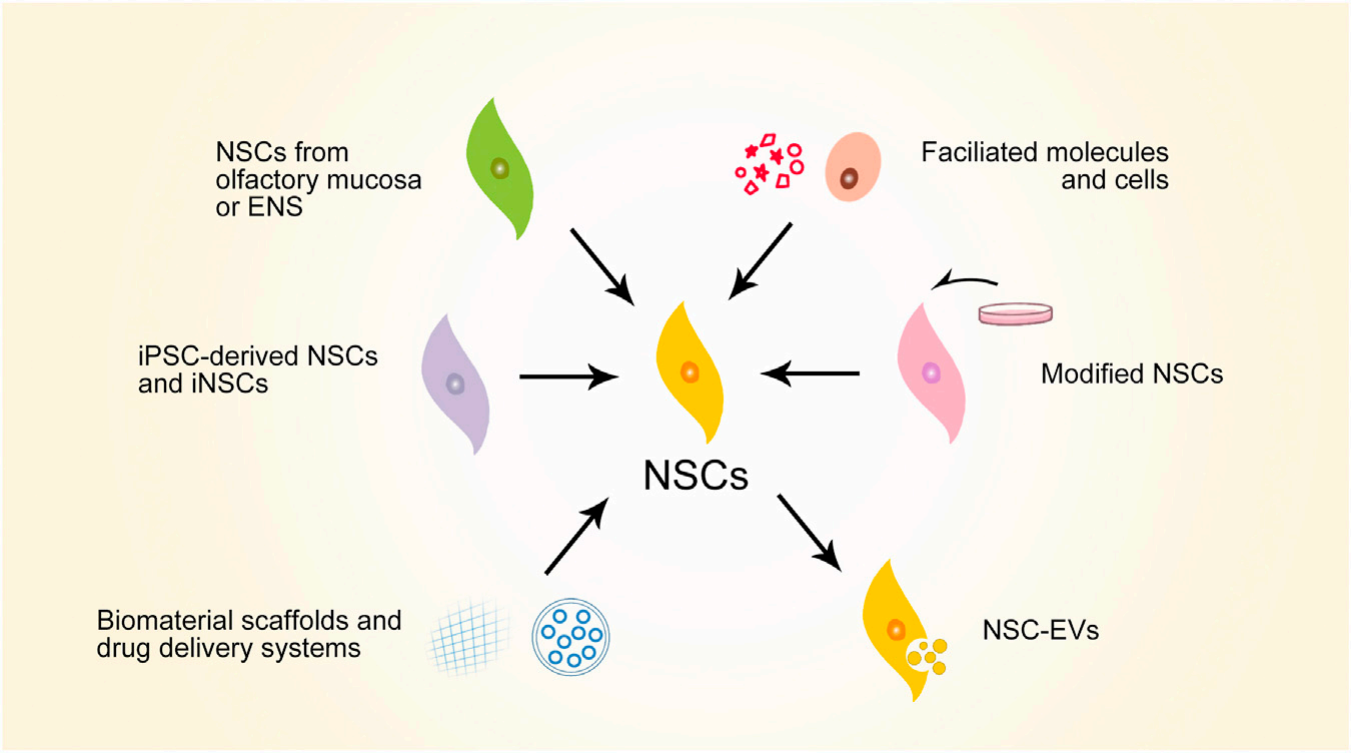
Schematic overview of major preclinical progresses of NSC-based strategies for SCI repair. NSCs, neural stem cells; ENS, enteric nervous system; iPSC, induced pluripotent stem cells; iNSCs, induced neural stem cells; NSC-EVs, NSC-derived extracellular vesicles.

Considering the therapeutic potential of NSCs, much promising preclinical progress has been made in the search for SCI repair strategies using NSCs (Figure 3). To summarize these strategies, iPSC-derived NSCs and iNSCs are alternative NSC sources that have been explored for treating SCI—these avoid ethical issues and are convenient for large-scale production. Olfactory mucosa and ENS are also considered for NSC extraction for autograft transplantation in the hope of lowering the level of post-transplantation rejection. Facilitated molecules and cells can enhance the therapeutic effects of NSCs. NSCs themselves can be modified for better reparative performance for SCI. NSC-derived extracellular vesicles (NSC-EVs) also have therapeutic effects for SCI. Biomaterials can facilitate NSCs in SCI repair, serving as scaffolds or drug delivery systems. Considering that various strategies have manifest efficacy in enhancing NSCs in various mechanisms, multiple strategies can be combined to achieve synergic effects. Typical studies representing this progress will be discussed in this section.

As well as the sources of NSCs used in studies, the types of SCI models are also provided because this information may influence the therapeutic outcomes of NSC-based therapies. Compared to rodent models, large animal and non-human primate models are more closely related to humans, making these models more useful for evaluating the therapeutic potential of treatment strategies for SCI repair in humans (Nardone et al., 2017). However, rodent models are still the most widely used animal models for SCI repair because large animal models require expensive care, rigorous regulatory requirements, and may raise ethical issues (Sharif-Alhoseini et al., 2017). As for different methods of generating SCI (contusion, compression, transection, and segment resection), transection models have been recommended because such models generate the more quantified and stable results of diffusion tensor imaging (DTI) and behavior assessments within four weeks after injury (Wang et al., 2014). However, contusion and compression models are also widely used because they better mimic the biomechanics and neuropathology of typical SCIs found in humans (Sharif-Alhoseini et al., 2017).

#### 4.1.1 iPSC-derived NSCs and iNSCs as alternative sources

iPSC-derived NSCs and iNSCs are convenient to culture on a large scale and are relatively free of ethical issues, especially when compared with NSCs from embryonic CNS tissue. Therefore, iPSC-derived NSCs and iNSCs are theoretically suitable for cell transplantation therapies.

The therapeutic effects of iPSC-derived NSCs for SCI have been verified by a 2019 meta-analysis (Ramotowski et al., 2019) which included studies from 2000 to 2018 using balloon compressed or contused thoracic SCI models in rats or mice. The iPSCs are derived from humans or mice. The overall data confirmed significantly better locomotor recovery at the 42-days mark than the control group. However, heterogeneity within the pooled studies did exist and more high-quality studies are needed to verify the therapeutic effects of iPSC-derived NSCs. Subsequent studies have supported the therapeutic effects of iPSC-derived NSCs. A study in 2021 generated human iPSCs from dermal fibroblasts and transplanted human iPSC-derived NSCs (hiPSC-NSC) into the lesion epicenter of the contusion T10 model in mice (Kong et al., 2021). Results showed decreased inflammatory levels, astroglial and neuronal differentiation fates, and reduced fibrotic tissue areas compared with the control group. When compared with the mesenchymal-stem-cell transplantation group, the transplantation of hiPSC-NSCs also had superior locomotor recovery *in vivo*. Therefore, the application prospects of iPSC-derived NSCs are hopeful for SCI repair.

However, despite these therapeutic effects, safety issues restrict the application of iPSC-derived NSCs—particularly tumorigenicity (Nagoshi et al., 2020). Researchers usually use immunosuppressants to reduce general adverse events such as rejection (Ramotowski et al., 2019). However, tumorigenicity remains a tricky problem after transplantation. In mouse models, hiPSC-derived NSCs formed tumors and motor function deteriorated after the 28th day after T10 contusion (Okubo et al., 2016). Hence, pretreated γ-secretase inhibitors (GSIs) were added before the transplantation to inhibit Notch signaling; the results indicated that this pretreatment can decrease the proliferation capability of hiPSC-derived NSCs and thus inhibit the overgrowth of hiPSC-derived NSCs after transplantation. Long-term motor function recovery was thus maintained. In addition to GSI pretreatment, inducible caspase systems are another way of preventing tumor formation. An orthogonal method has recently been designed to eliminate undifferentiated iPSC-derived NSCs (NANOG+) with AP20187 to prevent teratoma forming and to eliminate all iPSC-derived cell lineages with ganciclovir or AP21967 if any adverse events emerge (Martin et al., 2020). This orthogonal method had the obvious effect of preventing tumor formation in mouse models. However, whether this strategy can help with SCI repair remains to be determined.

Compared with iPSC-derived NSCs, iNSCs have the unique advantages of more cost-efficient preparation protocols and lower tumorigenic risk, making them suitable candidates for NSC-based therapy (Tian et al., 2018). However, fewer studies have used iNSCs to treat SCI than iPSC-derived NSCs—possibly because of the lack of consensual preparation protocols and authoritative iNSC profiles (Erharter et al., 2019). Moreover, evidence showed that the transplantation of iNSCs induced from human BJ fibroblasts did not significantly improve locomotor function in a thoracic contusion SCI model in mice (Braga et al., 2020). Therefore, the optimization of protocols for preparing iNSCs and innovation in the strategy of using iNSCs are essential to achieving better therapeutic effects for SCI. Although not many, there are modifications of iNSCs or biomaterial scaffolding to help iNSCs exert reparative potential for SCI; such strategies will be discussed below.

#### 4.1.2 Olfactory mucosa- or ENS-derived NSCs as alternative sources

The prominent advantages of NSCs extracted from olfactory mucosa or ENS are that they are easily accessed, can be used for autologous transplantation, and may thus reduce rejection reactions.

Current evidence suggests that NSCs from olfactory mucosa can facilitate SCI repair. The transplanted NSCs from rat olfactory mucosa survived and differentiated into neurons in the SCI lesion sites in a rat contusion SCI model at T10 level, with significant functional recovery after four weeks post-surgery (Muniswami et al., 2017). NSCs can be conveniently extracted from human participants instead of experimental animals. These sources of NSCs have proved their capabilities for significant, although limited, functional recovery in a rat contusion SCI model at T9–T10 level (Voronova A. et al., 2020).

ENSCs are another attractive candidate for autologous transplantation. In chicken embryos, ENSCs mostly differentiated into neurons and formed extensive connections in the anterior/posterior axis across the lesion sites in the spinal cord (Jevans et al., 2018). This provides preliminary evidence for the therapeutic potential of ENSCs. A recent 2021 study co-transplanted ENSCs with chondroitinase ABC to degrade inhibitory chondroitin sulfate proteoglycans (CSPG) content in the glial scars in the contused spinal cord of rats at T10 level (Jevans et al., 2021). The cavity area was thus reduced and projections through and past the lesioned area were reconstructed. However, the outcome of locomotor recovery was limited, indicating that ENSC-based strategies need to be further explored.

#### 4.1.3 Facilitated molecules and cells for NSC potentiation

The therapeutic potential of NSCs can be enhanced by the addition of supplementary materials, including certain molecules or cells. Transplantations of such combinations usually have superior reparative effects; their mechanisms vary with the specific materials added.

Many factors involved in various signaling pathways can be considered, especially those that target proliferation, migration, and the differentiation of NSCs. For example, erythropoietin (Epo) possesses anti-apoptotic, anti-inflammatory, and pro-vasculogenic functions, making it a possible candidate for treating SCI (Mofidi et al., 2011). Injecting Epo into a rat contusion SCI model at T10 level elevated oligodendrogenesis and neurogenesis from endogenous NSCs *in vivo* and *in vitro* (Zhang et al., 2018). As a result, locomotor recovery was significantly superior in Epo injection groups. Certain inorganic compounds, such as lithium, also showed the promotive effects of NSCs. Lithium chloride shows physiological effects on NSCs that promote their survival, proliferation, migration, and differentiation (Ferensztajn-Rochowiak and Rybakowski, 2016). Co-loading human NSCs derived from ESCs with lithium chloride prompted transplanted NSCs to migrate in a rostrocaudal direction and differentiate into neurons and astrocytes in a rat contusion SCI model at T10 level (Mohammadshirazi et al., 2019). Proliferation levels of endogenous NSCs were also elevated *in vivo*. Moreover, superior locomotor recovery was significant after only one post-transplanted week, which was quite astonishing.

Cells, as well as molecules, can be co-implanted with NSCs. The main options include Schwann cells and olfactory ensheathing cells (OECs). In a rat transection SCI model at T10 level, superior locomotor recovery has been witnessed when rat Schwann cells with enhanced expression of NT-3 and rat hippocampal NSCs with enhanced expression of Trk-3 were co-implanted (Wang et al., 2011). Moreover, co-implant rat hippocampal NSCs with rat Schwann cells can promote neuronal differentiation of NSCs, inhibit astroglia differentiation of NSCs, and secrete multiple neurotrophic factors, including BDNF and GDNF (Yu et al., 2017). Besides Schwann cells, OECs—the natural glial cells in the olfactory system—are another popular candidate as cograft cells, considering their potential to support neuronal survival and growth (Reshamwala et al., 2019). Rat OECs can inhibit necroptosis of rat hippocampal NSCs as well as increase their survival, proliferation, and differentiation, leading to significant motor and autonomous improvement in a rat contusion SCI model at T10 level (Wang X. et al., 2021).

#### 4.1.4 Genetically transfected NSCs for cell transplantation

Transfection is a powerful and commonly-used method of introducing exogenous genes or non-coding RNAs. Transfected NSCs can possess enhanced biological behaviors or additional therapeutic potential. For example, transfection of miR-124-containing plasmids into rat bone marrow-induced NSCs endowed iNSCs with promoted neuronal differentiation and inhibited astrocyte differentiation (Xu et al., 2012)—possibly because miR-124 has the capability of shifting the gene expression profile to induce neuronal differentiation (Cao et al., 2007; Makeyev et al., 2007). Consequently, decreased lesion cavity volume and improved functional recovery in a rat contusion SCI model at T9–T10 level have been observed (Xu et al., 2012). Transfection technology can also combine other strategies to achieve advanced reparative effects. Tropomyosin receptor kinase C (TrkC)-modified rat NSCs from hippocampal tissue, prepared by transfection, have worked in synergy with a NT-3-releasing fibroin scaffold in a rat SCI model at T9 level (Li G. et al., 2021). The interaction of TrkC and NT-3 promoted neurogenesis in an embryonic spinal cord. Moreover, the transfection of such compound biomaterial achieved superior survival and neuronal differentiation of modified NSCs. An organized neural network was rebuilt and significant locomotor function was observed.

The immortalization of NSCs can be achieved with the help of transfection technology. The survival, proliferation, and differentiation of NSCs can thus be enhanced, which are beneficial for SCI repair. Lentiviral transfection can introduce the c-mycER^TAM^ gene into NSCs, which expresses a fusion protein of the *c*-myc gene with a mutated estrogen receptor (Pollock et al., 2006). This fusion protein can response to synthetic 4-hydroxy tamoxifen and activate the proliferation of host cells, achieving a conditional induction of immortalization. With the help of this technology, an immortalized type of human NSCs from fetal spinal cords (SPC-01 cells) has been generated (Amemori et al., 2013). SPC-01 cells can modulate an immune microenvironment, with significantly downregulated TNF-α and NF-κB levels in a rat contusion model at T8 level (Karova et al., 2019). Furthermore, SPC-01 cells can increase the secretion of NGF and NT-3, decrease the secretion of BDNF and VEGF, and enhance the differentiation of motor neurons (Amemori et al., 2013). Transplantation of SPC-01 cells into a rat compression model at T8–9 level showed multiple reparative developments, such as enhanced axonal sprouting, reduced formation of glial scars, decreased size of cavity volume, enhanced gray matter preservation, and significant recovery of sensory and locomotor functions. Hence, the immortalization of NSCs is a promising technology in NSC-based therapies for SCI.

Considering that the hypoxic conditions in the SCI lesion sites hinder NSCs from exerting their reparative functions, NSCs can be modified to target or accommodate to hypoxic microenvironments. Evidence shows that the hypoxic precondition of rat cortical NSCs promoted their neuronal differentiation and neurotrophic-factor secretion (Fan W.-L. et al., 2018). As a result, significant motor and sensory improvement can be achieved in a rat contusion SCI model at T10 level. Moreover, with the help of hypoxia-responsive elements (HREs), NSCs can possess specific functions under hypoxic conditions. For instance, HREs can be conjugated with basic FGF (bFGF) cDNA sequence via adeno-associated virus 2-based expression vectors (Zhu et al., 2021). Transfected rat cortical NSCs can specifically express bFGF under hypoxic conditions, resulting in inhibited SCI-induced autophagy, increased neuronal differentiation, attenuated astrocyte differentiation, enhanced axonal sprouting, and significant locomotor recovery in a rat contusion SCI model at T9 level.

#### 4.1.5 NSC-EVs for SCI repair

NSC-EVs have been recently proven to possess therapeutic potential for SCI, mainly because of the contents inside NSC-EVs. 14-3-3t protein is one type of content which usually protects cells from stress, regulating cell cycles, protein trafficking, and steroid production (Aghazadeh and Papadopoulos, 2016). 14-3-3t inside NSC-EVs can interact with Berlin-1 to activate autophagy, leading to anti-inflammatory and anti-apoptotic effects of NSC-EVs (Rong et al., 2019a). The *in vitro* experiments found that NSC-EVs can activate autophagy, decrease neuronal apoptosis, and suppress nitric oxide production by macrophages. Besides 14-3-3t protein, various microRNAs and proteins are involved in NSC-EVs and are related to many important signaling pathways (Upadhya et al., 2020). Of these pathways, neuroprotection, anti-apoptosis, antioxidation, and anti-inflammation are their main functions. This forms the basis of the therapeutic potential of NSC-EVs. As supporting evidence, in a rat contusion model at T10 level, transplanting NSC-EVs from fetal mouse spinal cords showed attenuated neuronal death, suppressed neuroinflammation, and significant functional recovery (Rong et al., 2019b).

#### 4.1.6 Drug delivery systems and biomaterial scaffolds for NSC facilitation

##### 4.1.6.1 Drug delivery systems

Drug delivery systems can be deliberately designed to facilitate NSCs to exert therapeutic effects for SCI. Polymeric carriers are widely used drug delivery carriers which have hydrophilic shells which form micelle structures and hydrophobic cores to load drugs (Hwang et al., 2020). Taking polysialic-acid-based (PSA-based) micelles loaded with minocycline (MC) as an example, PSA attaches to the cell adhesion molecules of NSCs (Hildebrandt and Dityatev, 2015) while MC suppresses secondary injury processes, such as inflammatory, apoptotic, and oxidative injuries (Papa et al., 2013). Transplanting such micelles into a rat contusion SCI model promoted the recruitment of endogenous NSCs to the lesion sites, increased neuron regeneration, extended axons, reduced glial scars, and facilitated significant locomotor recovery (Wang et al., 2019). Besides polymeric nanocarriers, RNA-based nanomaterials have shown superior thermal stability, structural flexibility, and functional diversity (Guo, 2010). Packing RNA (pRNA) can form nanostructures via three-way junctions and bear small interference RNA (siRNA) targeting interested genes, such as *Lcn2*, a mediator of neuroinflammation (Braga et al., 2020). The injection of such particles (pRNA-siRNA) aims to provide an advantageous microenvironment for the subsequent transplantation of human BJ fibroblast-induced NSCs. This combined strategy showed a synergistic promotion of neuronal survival and histopathological improvement in thoracic-contused SCI mice at T12 level. Considering the convenience of manipulating RNAs, more diverse functions can be achieved and better reparative outcomes can be expected. In summary, appropriate drug delivery systems with certain bioactive molecules can potentiate or facilitate the therapeutic effects of NSCs, serving as a potential strategy for SCI repair.

##### 4.1.6.2 Self-assembly peptides scaffolds

Self-assembly peptides (SAPs) are being extensively explored as scaffolds for loading NSCs, which can self-assemble into nanofiber structures and bridge the lesion cavity in the injured spinal cord (Fichman and Gazit, 2014). One of the most prominent advantages of SAPs is the convenience of selecting and modifying the residues of SAPs to perform various functions. QL6 (K_2_ (QL)_6_K_2_) SAPs, for instance, can self-assemble into uniform nanofibers with β-sheet conformation, amphiphilic structure, and degradable characteristics (Zweckberger et al., 2016). Such material can optimize hostile microenvironments in SCI lesion sites. Injecting QL6 before the transplantation of mice subventricular NSCs increased survival and promoted the neuronal and oligodendrocyte differentiation of NSCs. The results showed reduced intramedullary cysts, increased tissue preservation, and superior locomotor recovery in a rat compression SCI model at C7 level. Besides QL6-containing SAPs, other SAPs—such as IKVAV- (Yin et al., 2021) and RADA16-containing (Zhai et al., 2020) SAPs—also showed potential to work in synergy with NSCs to treat SCI, due to the cell adhesion function given by their unique peptide sequences and modifications. By designing peptide and derivatives of SAPs, more varied functions can be achieved. Therefore, the applications of scaffolding SAPs to enhance the reparative effects of NSCs are promising.

##### 4.1.6.3 Collagen-based scaffolds

Collagen, a common component in ECM, is another source of tissue engineering. With the prominent advantages of biocompatibility and biodegradability, various types of collagen-based scaffolds have been designed for NSC-based therapies.

Functional collagen-based scaffolds can be prepared by crosslinking functional molecules to collagen. For example, linear ordered collagen scaffolds can crosslink with N-cadherin (LOCS-Ncad) (Liu W. et al., 2020). N-cadherin is a glycosylated transmembrane protein that regulates adhesion between cells (Hong and Brewster, 2006) and may promote neuronal differentiation via the Wnt signaling pathway (Maher et al., 2009). *In vitro* experiments verified that LOCS-Ncad scaffolds can increase the adhesion of rat meningeal NSCs onto the scaffolds and enhance neuronal differentiation (Liu W. et al., 2020). Transplanting LOCS-Ncad into a thoracic transection SCI model of rats recruited endogenous NSCs to the lesion sites and promoted their neuronal differentiation. Significant electro-physiological and locomotor recovery were also witnessed.

Collagen can be edited to be multifunctional. Current multifunctional collagen scaffolds that potentiate the therapeutic effects of NSCs are mainly combined with drug delivery systems to achieve multifunction. For example, exosomes from human umbilical cord-derived mesenchymal stem cells (MExos) can be retained by modifying collagen with a dual bio-specific peptide (Zhang et al., 2021). MExos, serving as paclitaxel (PTX) vehicles, can enhance the recruitment and migration of endogenous NSCs, while the loaded PTX can promote the neuronal differentiation of NSCs. The transplantation of such multifunctional collagen scaffolds showed not only the reparative effects of MExos and PTX but also superior functional recovery in a T8 transection SCI model of rats.

Besides macro-structured collagen scaffold, micro/nano-structured collagen scaffolds possess unique advantages for constructing delicate structures, facilitating certain behaviors of NSCs. For example, multichannel collagen scaffolds mimicked native multichannel neural conduits, promoting the recruitment of endogenous NSCs and enhancing the growth of axons in a T9 transection SCI model of rats (Sun et al., 2019). As a result, superior reparative capability has been witnessed within a suppressed inflammatory microenvironment, decreased cavity volume, reduced glial scars, and significant locomotor recovery.

Collagen scaffolds can incorporate electrospun fibers to form complex scaffolds, better supporting the behaviors of NSCs. Electrospun fibers are increasingly valued in the field of tissue engineering due to their capabilities of mimicking the native structure of ECM and guiding neural regeneration (Braghirolli et al., 2014; Schaub et al., 2016). Electrospun fibers can be deliberately arranged, modified, and can incorporate facilitatory factors, further extending the functions of the fibers. For instance, radially aligned poly (ε-caprolactone) fibers were created by electrospinning in the collagen matrix with loaded continuous gradients of stromal-cell-derived factor-1α (SDF1α) (Li et al., 2016). The radially aligned scaffolding mats provided topographic cues for mouse hippocampal NSCs while SDF1α provided chemotactic signals for NSCs. As a result, the transplantation of such scaffolds oriented the migration of NSCs from the periphery to the center of the lesion sites, providing a promising way of guiding neural regeneration *in vitro*. Compared with specifically-aligned electrospun fibers, injectable magnetic electrospun fibers are an important advancement for providing a more convenient way to change the topography *per se*. Magnetism can be achieved by incorporating superparamagnetic iron oxide nanoparticles (SPIONs) into poly-l-lactic acid (PLLA) fibers (Johnson et al., 2019). Such fibers suspended in collagen can be oriented by the external magnetic field and locked by solidifying the collagen matrix to maintain proper orientation. *In vitro* experiments confirmed that such fibers can promote neurite alignment and length in neurons from rat dorsal root ganglions. However, this technology has not yet been applied to NSC-based therapy. With the improvement of the physicochemical properties of electrospun fibers and the innovation of fabrication strategies, electrospun-fiber-added scaffolding may exert more evident and diverse effects in assisting NSC-based therapies for SCI repair.

##### 4.1.6.4 Hydrogel-based scaffolds

Hydrogel is a 3D network of hydrophilic polymers that can hold water and maintain structure in the meanwhile, serving as another suitable candidate for constructing scaffolds (Majee, 2016). These scaffolds can potentiate the reparative efficacy of NSCs, which will be discussed in this section. Since collagen-based scaffolds were discussed in the previous section, this section will exclude collagen hydrogel scaffolds.

Among the many materials for fabricating hydrogel, gelatin methacrylate (GelMA) has attracted much attention, mostly because it shares similar physicochemical characteristics with native nerve tissues, and stiffness can be regulated to control the proliferation and differentiation of loaded stem cells (Wang et al., 2010). When photoencapsulated with mouse iPSC-derived NSCs, GelMA potentiated neurite outgrowth and enhanced neuronal differentiation in a mouse transection SCI model at T9–T10 level (Fan L. et al., 2018). Moreover, polyethylene glycol-gelatin methacrylate (PEG-GelMA) can be used to generate biomimetic hydrogel by 3D printing (Koffler et al., 2019). 200-μm microchannels mimicked axonal tracts in the white matter, while solid material mimicked gray matter. The gel was then shaped according to precise shapes and dimensions in the SCI lesion cavities of a rat transection SCI model at T3 level. Injecting this gel loaded with rat spinal NSCs into SCI rat models showed improved survival of NSCs and axonal regeneration. Moreover, regenerated axons aligned along the axis of host axons, mimicking well the native structures in the spinal cord. As well as 3D hydrogel, 4D dynamic hydrogel can be generated using hybrid GelMA-microcapsule hydrogel (HGMH) with sinuate microwrinkles according to asymmetric water swelling (Chiang et al., 2021). Poly- (lactic-co-glycolic acid) (PLGA) microcapsules, which were aligned on HGMH, loaded with NT-3 arrayed into triangle pattern by dielectrophoresis and produced a gradient of NT-3 to modulate cell differentiation and growth. Transplantation of such materials promoted endogenous NSCs to migrate to the lesion sites, increasing neuronal differentiation in a rat hemisection SCI model at T8 level.

In addition to GelMA, hydrogel used in SCI therapy can also be derived from decellularized tissue matrix to mimic the native microenvironment. Research found that *in situ* injection of porcine decellularized spinal cord matrix hydrogel (DSCM-gel) promoted viability, proliferation, migration, neuronal differentiation, and the synapse formation of rat hippocampal NSCs *in vitro* (Xu et al., 2021). ECM protein may contribute to the regulation of the integrin expression profile and AKT/ERK-related signaling pathways in NSCs. Furthermore, *in vivo* experimentation has shown that DSCM-gel provided a preferable microenvironment for recruiting NSCs and regenerating axons, leading to significant locomotor recovery. In addition, DNA has recently been used to fabricate hydrogel. Its prominent advantage is its high permeability, which has usually been overlooked. The transplantation of a highly-permeable DNA hydrogel showed enhanced proliferation and differentiation in both implanted and endogenous NSCs, with new formation of a neural network in a rat transection SCI model at T10 level (Yuan et al., 2021). The hindlimb function was basically recovered with signal transduction by renascent synapses in the lesion sites.

Hydrogel can be designed to possess high conductivity, thus facilitating nerve regeneration. Highly conductive hydrogel can be generated with polypyrrole (PPy) crosslinked by tannic acid (Zhou L. et al., 2018). *In vitro* experiments found that such hydrogel can promote neuronal differentiation and inhibit astrocyte differentiation of mouse hippocampal NSCs. Transplantation of such material into a rat hemisection SCI model constructed a highly conductive bridge into the lesion sites at T9–T10 level. Results showed an enhanced activation and neurogenesis of endogenous NSCs in the lesion sites and significant locomotor recovery.

Hydrogel can also be multifunctional, better exerting facilitative functions for NSCs. Multifunctionality can be achieved in many ways, including integrating functional molecules (Wang Y. et al., 2020) and combining with various drug delivery strategies (Wang et al., 2018). For example, modified hydrogel with SAPs coupled with these materials with certain growth factors generated a special type of SAP hydrogel (Liu H. et al., 2020). Such SAP hydrogel bridges cavities in SCI sites and possesses multiple functions of each construction module in a rat T9 transection SCI model. As a result, the proliferation and neuronal differentiation of endogenous NSCs have been observed, along with enhanced maturation, myelination, and the formation of interconnection with lesioned descending corticospinal tracts, leading to locomotor recovery and electrophysiological improvement.

However, the intrinsic swelling capabilities of hydrogels may lead to the content exuding and thus hindering the implementation of a complex hydrogel-based strategy. The latest approach constructed hydrogels with oxidized dextran and hyaluronic-hydrazide, which manifested a reduced swelling ratio compared to conventional hydrogel (Huang F. et al., 2021). When loaded with BDNF that was encapsulated with tannic acid-modified PLGA, the hydrogel manifested high electrical conductivity, improved stability, and an extended release time of BDNF from the matrix. Such material promoted neuronal differentiation and inhibited astrocyte differentiation of rat subventricular NSCs *in vitro*, showing a promising potential for SCI repair.

#### 4.1.7 Complex strategies to achieve synergy

Complex strategies can be explored given the various mechanisms for enhancing the therapeutic potential of NSCs and the manipulable capabilities of biomaterials. The components of combined strategies may mechanically compensate each other, achieving synergistic effects. For example, human iPSC-derived NSCs and activated rat Schwann cells can be loaded onto polycaprolactone (PCL) electrospun fibers (Zhou X. et al., 2018). iPSC-derived NSCs provided NSC sources, along with endogenous NSCs. The activated Schwann cells can provide remyelination capabilities and secrete various neurotrophic factors. The PCL fibers provide a biocompatible and biomimetic microenvironment for supporting the loaded NSCs. As a result, the volume of lesion cavity was reduced and superior locomotor recovery was found in a rat T10 transection SCI model. Nevertheless, no strategies have yet achieved complete recovery after SCI in animal models. Therefore, more diverse strategies and effective combinations are required.

### 4.2 Attempts in clinical trials

Table 1 lists completed or terminated clinical trials of NSC transplantation therapy for SCI treatment to date. When compared with the more abundant preclinical tests, very few clinical trials have been completed for studying NSC transplantation therapy for SCI repair. These trials all used similar transplantation methods of injecting human NSCs into intramedullary spaces. They are all in relatively juvenile phases and are mainly designed to provide safety profiles.

**TABLE 1:** Summary of outcomes of clinical trials of using NSCs to treat SCI.

Year	Trial registry No.	Investigators	Type of trial and stage	Targeted patients	Transplantation source of NSCs	Data collection	Follow up period	Outcomes
2015 (Shin et al., 2015), completed	KCT 0000879	Shin et al	Cohort study, phase I/IIa	AIS A or B cervical SCI of traumatic etiology, 19 in the experimental group, 15 in the control group	Human fetal CNS-derived NSCs (hNSCs)	AIS neurological examination, SSEP, MEP, MRI, pain and spasticity assessments, AMS, ASS, UEMS, LEMS	12 months	Safe and well-tolerated, modest neurological benefit (5/19 and 1/15 showed AIS grade improvement)
2015, completed	NCT 01321333	Huhn et al	Cohort study, phase I/II	12 patients with AIS A, B, or C thoracic SCI	NSCs derived from human CNS (HuCNS-SC^®^, Stemcells, Inc., Newark, CA)	AE, AIS grade improvement	12 months	Not provided
2016, terminated (business decision)	NCT 01725880	Huhn et al	Observational study	12 patients undergone thoracic HuCNS-SC^®^ transplantation	HuCNS-SC^®^	AIS grade improvement	48 months	Not provided
2018 (Curtis et al., 2018), primary completion	NCT 01772810	Curtis et al	Cohort study, Phase I	4 patients with s AIS A thoracic SCI	Human spinal cord derived NSCs (NSI-566)	AE, motor function, life quality, postoperative changes	60 months	Well tolerated, 3 patients showed modest neurological improvement
2019 (Levi et al., 2019), terminated (business decision)	NCT 02163876	Levi et al	Single blinded, RCT, Phase II	12 patients with complete motor C5-C7 SCI (AIS A or B)	HuCNS-SC^®^	AE, UEMS, GRASSP, MRI, tacrolimus	12 months	Reliable safety and feasibility for HuCNS-SC transplantation, but underpowered *a priori* futility analysis

KCT, trials were registered with Clinical Research Information Service (CRIS); NCT, trials were registered on ClinicalTrials.gov; AIS, American Spinal Injury Association Impairment scale; SSEPs, Somatosensory evoked potentials; MEPs, motor evoked potentials; MRI, spinal cord magnetic resonance imaging; AMS, ASIA, motor scores; ASS, ASIA, sensory scores; AE, adverse events; UEMS, upper extremity motor score; GRASSP, graded redefined assessment of strength, sensibility, and prehension.

As for safety outcomes, the enrolled participants all tolerated the transplantation surgery well. The transplantation of human NSCs showed no association with greater risks of serious adverse neurological effects and complication profiles, compared with SCI patients without transplantation therapy. As for secondary outcomes such as American Spinal Injury Association Impairment Scale (AIS)-grade changes and other neurological assessments, some moderate improvement was witnessed. These results held promising prospects for NSC transplantation therapies, albeit with limited statistical power and imperfect control designs.

Therefore, although the therapeutic effects of NSC transplantation cannot be determined for SCI, preliminary conclusions can be drawn that this transplantation strategy is well-tolerated and relatively safe. Subsequent phases of clinical trials are needed to further escalate cell doses and verify the functional improvement of NSC transplantation therapy for SCI repair.

## 5 Major obstacles for application of NSCs

Although extensive studies have been conducted concerning NSCs’ application to SCI repair—especially in preclinical tests—complete recovery is still a remote objective. The major reasons are listed as follows.

Firstly, safety concerns about iPSCs restrict their application in SCI repair. Current sources of NSCs are largely restricted to extraction from embryonic tissue and involve ethical issues and low efficacy of production. Theses drawbacks can be overcome by iPSC-derived NSCs; iPSC-derived NSCs are attracting much attention as substitutes for embryonic-derived NSCs. Although many studies have confirmed the reparative effects of iPSC-derived NSCs, tumorigenicity remains a major problem that hinders their application (Ramotowski et al., 2019). There are many strategies that aim to suppress tumor formation, such as γ-secretase inhibitor (Okubo et al., 2016) and induced caspase systems (Martin et al., 2020). However, these are highly expensive and their effects for SCI repair have not been definitely verified. Hence, a wide application of iPSC-derived NSCs has not yet been achieved.

Secondly, the differentiation routes of NSCs are mostly unwanted. SCI leads to the loss of neuronal and neuroglial cell lineages. Therefore, the ideal differentiation fates of NSCs are both neuronal and neural glial fates. Additionally, newly regenerated axons should form connections and functional circuitry with existing neurons. However, while endogenous NSCs normally differentiate into astrocytes and oligodendrocytes, leading to glial scar formation, exogenous NSCs face challenges of poor survival rates as well as unwanted differentiation fates. Researchers have already focused on altering the intrinsic differentiation fates of NSCs (Hooshmand et al., 2017; Wang M. et al., 2020). However, translating such achievements into animal models has not yet matured. Moreover, current types and proportions of differentiated neuronal cells from NSCs cannot be manipulated by researchers. Evidence has shown that differentiated NSC neurons are dominantly GABAergic phenotypes, which deviate from neuronal constitutions in the native spinal cord (Shechter et al., 2007). Furthermore, motor axonal regeneration in SCI lesion sites has failed to promote motor functions *in vivo*, although the growth of motor axons was indeed stimulated (Lu et al., 2012). These results suggest the complexity of SCI repair and the necessity of delicately controlling the differentiation fates of NSCs.

Thirdly, the assessment of the reparative effects of therapies cannot comprehensively represent the conditions in the injured spinal cord. Spinal cords have motor, sensory, and autonomous functions. However, while most studies assessed motor recovery using various tests and gave straightforward results, sensory and autonomous assessments were mostly overlooked. It may be that tests to assess sensory and autonomous functions are more expensive or bothersome. Alternatively, results may not be obvious or are difficult to analyze. Consequently, questions remain regarding the effects of NSCs to repair sensory and autonomous losses after SCI.

Fourthly, most preclinical tests adopt rodent animal models, which deviate from human models. Theoretically, data from large animals and nonhuman primates better simulate conditions in humans. However, these models usually generate uncommon clinical SCI type or reproduce inconsistently (Cheriyan et al., 2014), hindering the conduct of studies. Nonetheless, promising yet preliminary results have been found in large animals and nonhuman primates. For example, Kobayashi et al. transplanted iPSC-derived NSCs into marmoset models, which promoted functional recovery after contusive SCI at C5 level of (Kobayashi et al., 2012).

A fifth reason why recovery is remote is a lack of high-quality data in clinical trials, a serious problem that hinders clinical translation of NSCs for use in SCI repair. In the field of clinical trials of stem cell-based therapies for SCI, NSCs are less popular than MSCs, largely because of the former’s limited availability (Silvestro et al., 2020). Unlike MSCs, which can be extracted from bone marrow, adipose tissue, and umbilical cords, NSCs used in clinical trials were all derived from human CNS tissue (see Table 1). This restricts the application of NSCs in the clinical translation and can raise ethical problems. Furthermore, the dose of NSCs for transplantation may be relatively high (Hu et al., 2021); the difficulty of obtaining enough NSCs may thus obstruct recruitment of more participants. In addition, the statistical credibility and quality of the existing studies were not sufficient to draw convincing conclusions regarding the application value of NSC transplantation strategy. Business factors may also hinder the conduct of clinical trials. As shown in Table 1, two out of five such trials have been terminated early because of business decisions rather than safety concerns.

Finally, many mysteries remain to be solved regarding NSCs in SCI repair. A limited understanding of NSCs has restricted thorough efficacy assessment and innovative strategy design in SCI repair. For example, whether endogenous NSCs in the adult spinal cord are ependymal cells is still contested (Xue X. et al., 2021). Consequently, current strategies of identifying and stimulating NSCs may need refinement. In any case, the specific signals that regulate the survival, proliferation, migration, and differentiation of NSCs remain elusive. Adverse events may hinder further applications, since current strategies are utilizing signaling pathways that target many physiological processes, such as Notch (Wang M. et al., 2020) and Wnt pathways (Li et al., 2020b).

## 6 Future perspectives

Firstly, directly induced NSCs and iPSC-derived NSCs are recommended over more frequently-used NSCs from embryonic tissues out of ethical and efficacy concerns. iNSCs and iPSC-derived NSCs are especially suitable when a large quantity of NSCs are needed, such as in clinical trials. While iPSC technology is more popular than induction technology, second-generation reprogramming—which allows direct conversion of somatic cells—provides an even faster, safer, and more cost-efficient way to prepare NSCs (i.e., iNSCs) (Erharter et al., 2019). Nevertheless, safety issues regarding both iNSCs and iPSC-derived NSCs in treating SCI remain to be solved. Innovative strategies for suppressing tumor formation are needed in this field. Moreover, the reparative efficacy of implementing such strategies in SCI repair needs further verification.

Secondly, differentiation control of NSCs in the spinal cord is needed. Current results have showed that transplanted NSCs mostly differentiate into astrocytes instead of neurons and oligodendrocytes (Kameda et al., 2018); this restricts the regenerative effects of NSCs. A more detailed understanding of the pathophysiology of SCI and the differentiation mechanisms of NSCs may help with strategy design to achieve the controlled differentiation of NSCs. NSCs may differentiate into and replenish cells damaged in SCI.

Thirdlly, *in vivo* assessments of NSC efficacy need refinement. While the structures and functions of native spinal cords are complex, current *in vivo* assessments are mostly restricted to immunohistochemical examinations and locomotor function assessments. Such assessments only provide a cursory understanding of SCI repair effects. Other aspects, such as sensory and autonomous recovery, are deliberately overlooked due to the limitation of the assessment methods. Hence, more thorough assessments are needed to provide a better understanding of the efficacy of NSC-based strategies. Novel strategies can be designed to directly improve the poorly repaired functions.

More diverse transplantation strategies should be adopted for clinical trials. Compared with abundant novel transplantation strategies in preclinical tests, current clinical trials all use direct NSC transplantation. A meta-analysis in 2020 showed that transplanting only NSCs in animal models yields only limited SCI repair (Qian et al., 2020). Considering that some strategies in animal models have shown promising results, more diverse strategies can be considered for application in clinical trials, such as a combination of neuroprotective factors, drug delivery systems, and/or scaffolds. However, strategies that use more than NSCs will introduce additional risks. Hence careful evaluation of the physiopathological basis of adopted strategies is needed.

The idea of NSC-based strategies can be extended to include more primitive neuroepithelial stem cells (NESCs). The current extraction of NSCs for transplantation may impair their intrinsic self-renewal and neurogenic properties, especially when NSCs are cultured as neurospheres (Reynolds and Rietze, 2005). NESCs, on the other hand, have better stem cell properties. NESCs extracted from spinal cord primordia have the capability of remaining adhesive and neurogenic in the long run, even without genetic immortalization (Dell'Anno et al., 2018). The *in vitro* experiments further proved the capability of NESCs to extend axons as far as extremities and restore significant physical functions. Therefore, NESCs may be superior to NSCs in retaining stem cell properties, and are thus promising for SCI repair.

In summary, NSCs serve as an important constituent of cell transplantation therapy for SCI. Their therapeutic potential establishes the basis of NSC-based therapies. Many strategies have been designed in preclinical tests with promising results. However, more work is needed to achieve full recovery and translate into clinical progresses.
